# News for a New Year

**DOI:** 10.20945/2359-3997000000204

**Published:** 2020-03-04

**Authors:** Marcello D. Bronstein

**Affiliations:** 1 Hospital das Clínicas Faculdade de Medicina Universidade de São Paulo São Paulo SP Brasil Editor-Chefe da Archives of Endocrinology and Metabolism, Chefe da Unidade de Neuroendocrinologia, Serviço de Endocrinologia e Metabologia, Hospital das Clínicas , Faculdade de Medicina da Universidade de São Paulo (HCFMUSP), São Paulo , SP , Brasil

Dear colleagues,

A new year begins for the *AE&M* and we have some important information to share.

1. Two new associate editors for the area of Obesity and Metabolic Disorders are joining us: Dr. Simone Van der Sande Lee and Dr. Bruno Halpern, who will face the arduous mission of replacing Dr. Erika Parente and Prof. Rogerio Friedman, leaving their positions due to other commitments assumed. Welcome Simone and Bruno to your new task!
2. As already stated in the Journal’s instructions to the authors, only Brazilian manuscripts with CAAE number (Certificate of Presentation for Ethical Consideration) generated at Plataforma Brasil will be considered for review ( 1 ). I believe that, unfortunately, many important articles will no longer be evaluated, but this measure will certainly improve the level of our Journal.
3. Finally, I hope that everyone has accepted the absence of the print edition, a general trend in medical journals. This measure undoubtedly streamlines, reduces costs and should, therefore, be maintained.

With my best regards.
